# Prevalence and Spatial Distribution of *Cephenemyia stimulator* in Roe Deer (*Capreolus capreolus*) from the North of Spain and Portugal

**DOI:** 10.3390/insects16030274

**Published:** 2025-03-05

**Authors:** Néstor Martínez-Calabuig, Madalena Vieira-Pinto, Ana Saldaña, Rosario Panadero, José Aranha

**Affiliations:** 1 INVESAGA Group, Department of Animal Pathology, Faculty of Veterinary, University of Santiago de Compostela, Avda Carballo Calero, s/n, 27002 Lugo, Spain; nestor.martinez.calabuig@usc.es (N.M.-C.); anasaldana.ruiz@usc.es (A.S.); rosario.panadero@usc.es (R.P.); 2 Veterinary and Animal Research Centre (CECAV), University of Trás-os-Montes and Alto Douro (UTAD), 5001-801 Vila Real, Portugal; mmvpinto@utad.pt; 3 Associate Laboratory for Animal and Veterinary Sciences (AL4AnimalS), University of Trás-os-Montes and Alto Douro (UTAD), 5001-801 Vila Real, Portugal; 4 Department of Veterinary Science, University of Trás-os-Montes and Alto Douro (UTAD), Quinta de Prados, 5001-801 Vila Real, Portugal; 5 Instituto de Biodiversidade Agraria e Desenvolvemento Rural (IBADER), Campus Terra, s/n, 27002 Lugo, Spain; 6 Centre for the Research and Technology of Agro-Environmental and Biological Sciences (CITAB), University of Trás-os-Montes and Alto Douro (UTAD), Quinta de Prados, 5000-801 Vila Real, Portugal; 7 Institute for Innovation, Capacity Building and Sustainability of Agri-Food Production (Inov4Agro), University of Trás-os-Montes and Alto Douro (UTAD), Quinta de Prados, 5000-801 Vila Real, Portugal; 8 Department of Forestry Sciences and Landscape Architecture (CIFAP), University of Trás-os-Montes and Alto Douro (UTAD), 5001-801 Vila Real, Portugal

**Keywords:** *Cephenemyia stimulator*, nasopharyngeal myiases, roe deer, oestridae, *Capreolus capreoulus*, Spain, Portugal

## Abstract

Since the first documented case of *Cephenemyia stimulator* in Spain in 2001, this myiasis has experienced rapid expansion, with the first case also detected in 2021 in Portugal. Between January 2020 and October 2024, a study was carried out in northern Spain (Cantabria, Galicia, País Vasco and Principado de Asturias) and Portugal (Bragança, Viana do Castelo and Vila Real) to evaluate the presence and distribution of this myiasis in these areas. The prevalence of infestation was 76.6 ± 4.72% (95% CI) and 38.78 ± 13.78% (95% CI), with a mean intensity of 62.27 ± 104.40 and 37.74 ± 36.84 larvae per animal in Spain and Portugal, respectively. Regarding age, larval intensity was significantly higher in young animals (109.76 ± 148.59) than in adults (32.22 ± 39.01) and old animals (33.64 ± 34.21) (*p* < 0.05). These results reflect a large increase in the prevalence and importance of younger animals in the spread of the disease. Therefore, it is very important to control and manage populations, focusing on the youngest animals.

## Simple Summary

This study aims to address the current situation of nasopharyngeal myiasis due to *Cephenemyia stimulator* (Clark) in roe deer from northern Spain and Portugal. This is the first epidemiological study about the situation of this myiasis in roe deer in Portuguese territory. The prevalence obtained in Portugal was 38.78%, with a larval intensity of 37.74 ± 36.84. Furthermore, this study reveals the highest prevalence (76.64%) and larval intensity (62.27 ± 104.40) in roe deer in Spain found to date. This parasite is a dipteran that deposits its larvae in the snout of the roe deer, and over a period of 6 to 10 months, these develop in the nasopharyngeal cavity, going through three larval stages. The larvae have hooks and spines that damage the nasopharyngeal mucosa of the roe deer, promoting secondary infections. In addition, in their last stage, they can reach a size of about 30 mm, causing many problems in breathing and ingesting food, especially due to their preferential location in the pharyngeal recesses. For this reason, it is necessary to establish effective control for this disease since high parasite loads can pose a risk to the survival of the animals.

## 1. Introduction

The larvae of flies of the Oestridae family parasitise most ungulates in a more or less specific way. In the case of the roe deer (*Capreolus capreolus*), the genus that primarily parasitises is *Cephenemyia* (Latreille) [1]. *Cephenemyia* larvae develop exclusively in cervids of the subfamily Cervinae and Odocoileinae and are only found in the Holarctic region. The adult specimens of this genus are similar to bumblebees, and like some other oestrids, they deposit larvae directly on the animal’s snout, which subsequently invade the nasal and pharyngeal cavity [2,3].

There are three types of larval stages, and the larvae have oral hooks and spines to facilitate their adherence and advancement through the nasal and pharyngeal cavities in addition to avoiding being expelled by the host’s defence mechanisms (coughing, sneezing, etc.) [4,5]. The size of the larvae ranges between 1 and 3 mm for larvae 1 (L1s), 3 and 13 mm for larvae 2 (L2s) and up to 30 mm for larvae 3 (L3s) [2].

L1s are deposited together with a dense fluid that favours their adhesion and prevents their desiccation. They quickly migrate to the nasal cavity, where they can remain in diapause during the cold winter months to avoid completing their development when environmental conditions are unfavourable for their survival [6]. When temperatures increase, they resume the cycle, and a large number go towards the pharynx where they enter into the mucosa recesses and develop into L2s and L3s. As the larvae grow, the mucosa becomes inflamed, giving rise to cavities with a large number of larvae, which are called retropharyngeal sacs. Their development is asynchronous to avoid competition between the larvae themselves [7]. When L3s have completed their growth, they carry out a reverse path to that taken by L1s, exiting through the nasal passages through the sneezes of the roe deer [8,9]. Once in the ground, L3s bury themselves since they are lucifuge and form a puparium of chitinous consistency that protects the larvae. If weather conditions are favourable, flies will emerge in about 2–3 weeks [10]. Adult flies lack mouthparts so they do not feed, and once fertilised, females seek shelter to expend as little energy as possible and be able to complete the incubation and hatching of the larvae [6,9]. Females are attracted by the smell and air exhaled by the host and deposit between 30 and 50 L1s in different hosts to guarantee the survival of the species [8,9].

*Cephenemyia* can cause health problems in roe deer in both its adult and larval stages. The flight of the flies increases the level of alertness, stress and nervousness of the deer, which run and shake their heads to try to free themselves from the dipteran, causing a decrease in food intake and use. In this way, in certain areas, they avoid going out to open areas around midday when fly activity is greater [9,10,11]. Also, the larvae cause irritation and erosion of the mucosa due to the action of their oral hooks and spines. In addition, the larvae secrete proteases that also contribute to damaging the mucosa, favouring secondary bacterial infections. Furthermore, the presence of larvae in the nasal and pharyngeal cavities causes sinusitis with coughing, sneezing, nasal discharge and difficulty in breathing and ingesting food [9,10,11,12]. The presence of between 30 and 80 mature larvae can have a serious impact on the health of the roe deer since dyspnea and dysphagia cause their body condition to worsen, and they become immunosuppressed, which can cause mortality, as well as being more susceptible to being preyed on by a predator [9,11,13].

In European roe deer, this myiasis is very prevalent, especially in Central Europe [14]. In Spain, the first reported case was in 2001 in the Province of Ciudad Real due to the importation of roe deer from France [15]. However, it seems that it remained an isolated incident, and it was in Asturias in that same decade when the first cases were seen, and their dispersion began [9,16].

In the case of Portugal, the first reported case was in 2021 in a roe deer hunted in Trás-os-Montes (NE Portugal), an area that is only 30 km from the northwest of Spain. Therefore, the most probable explanation for the entry of this myiasis in Portugal may be due to the natural expansion of Spanish roe deer populations [17].

## 2. Materials and Methods

### 2.1. Area of Study and Animals

This study was carried out between January 2020 and October 2024 in 4 autonomous communities in northern Spain (Cantabria, Galicia, País Vasco and Principado de Asturias) and 3 districts in northern Portugal (Bragança, Viana do Castelo and Vila Real). Samples were obtained from all months since they came from hunting, deaths due to being run over and other causes.

This study area is characterised by having a temperate climate with temperatures between 0 and 18 °C. Summer is mild in the Cantabrian region and is somewhat drier in Galicia and northern Portugal. The relative humidity is high, with numerous storms throughout the year [18,19].

This area of the Iberian Peninsula is characterised by a domain of deciduous hardwood forests (Beech (*Fagus sylvatica*), oak (*Quercus robur*) and mixed deciduous forests, with *Quercus pyrenaica* and *Quercus faginea* predominating in the south of Galicia and north of Portugal [20,21].

In certain places of the study area, the roe deer shares habitats with super-predators, such as the wolf, which has a high number of packs in the northwest of the peninsula [17] and other cervids that can compete for food resources [22].

**Figure 1:**
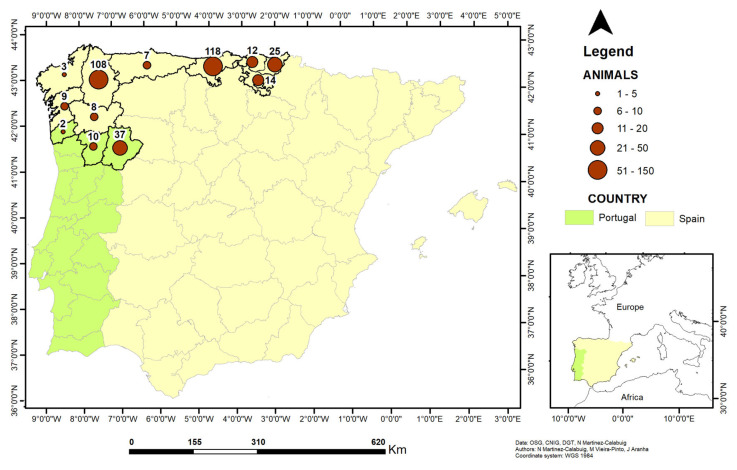
Distribution of sampled roe deer.

A total of 353 roe deer heads (304 from Spain and 49 from Portugal (Figure 1)) were collected from hunted animals and roadkill and subsequently frozen and stored at −20 °C. Each animal was identified with a file that reflected the date and cause of death and the municipality where it died. The animals were classified into 3 age ranges (<2 year; 2–6 year; and >6 year (Table 1)) based on the number, shape and wear of the teeth, according to Høye [23].

### 2.2. Larval Collection and Identification

**Figure 2:**
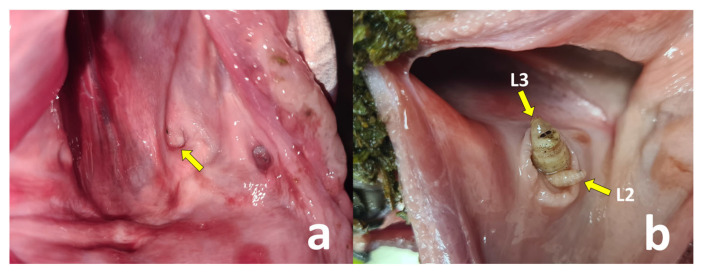
Pharyngeal recesses of a healthy roe deer (**a**) and of an infested roe deer with inflamed mucosa and L3 and L2 of *C. stimulator* inside (**b**).

The roe deer were examined following the same procedure as Martínez-Calabuig [6]. First, the pharyngeal region was accessed, where the absence or presence of dilation of the pharyngeal recesses and *Cephenemyia* larvae could be observed (Figure 2).

**Figure 3:**
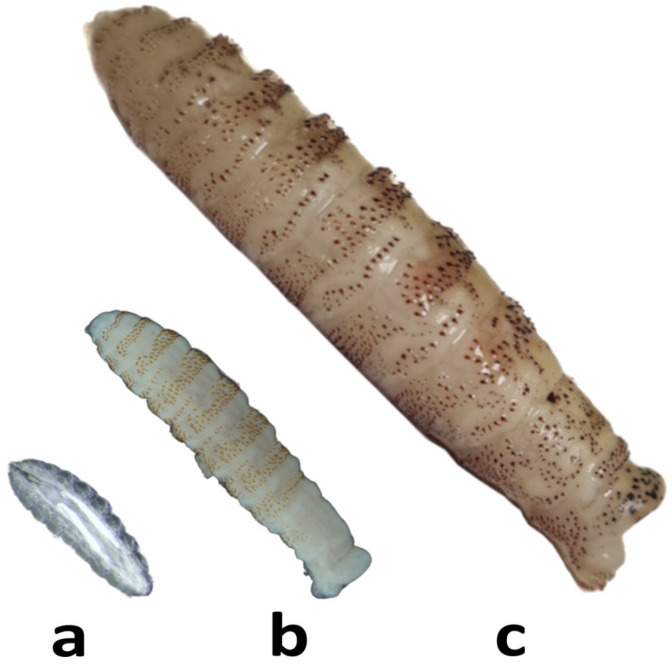
(**a**) L1, (**b**) L2 and (**c**) L3 of *Cephenemyia stimulator*.

Next, the nasal cavity was accessed. A detailed inspection and subsequent head washing were carried out over a filter mesh where the L1s were mainly collected. This process is especially important for the heads obtained during winter months since they would only house first-stage larvae in the nasal cavity. All the larvae that were collected were washed in physiological saline solution and classified according to their larval stage into L1s, L2s and L3s (Figure 3). For their morphological identification, the Zumpt key [2] was followed by studying the spinulation pattern, shape of the antennal lobes and respiratory plates. They were stored frozen at −20 °C or in 70° ethanol.

### 2.3. Statistical Analysis

We performed the Student *t*-test to analyse the statistical significance of the differences between the mean values calculated for each variable across different geographic locations, the genus of the animals and their age [24].

### 2.4. GIS Project

We used the GIS software QGIS (open source, version 3.28.6-Firenze, code revision 868c9fa03b) and ArcGIS 9.7 (commercial) to create the GIS project (administrative boundaries) that supports this research as well as the spatial representations of the variables under study (number of sampled animals) [25].

## 3. Results

A total of 252 of 353 (71.39 ± 4.72% (95% CI)) roe deer harboured *Cephenemyia* larvae (n = 15,226) in the nasopharyngeal cavity, with a mean intensity of 60.42 ± 101.06 larvae per animal. After morphological identification, 12,541 were classified as L1s, 880 as L2s and 1805 as L3s.

Divided by country, in Spain, there were 233 of 304 (76.64 ± 4.76% (95% CI)) infected, with a mean intensity of 62.27 ± 104.40, and in Portugal, there were 19 of 49 (38.78 ± 13.78% (95% CI)), with a mean intensity of 37.74 ± 36.84. The higher prevalence in Spain than in Portugal was statistically significant (*p* < 0.001), although the larval intensity was not (*p* > 0.05).

Within the Spanish autonomous communities, the highest prevalence was obtained in Galicia (91.41 ± 4.87% (95% CI)), followed by País Vasco (86.27 ± 9.54% (95% CI)), Principado de Asturias (85.71 ± 28% (95% CI)) and Cantabria (55.93 ± 9% (95% CI)). These differences in prevalence were only significant between animals from Galicia and País Vasco compared to Cantabria (*p* < 0.001).

Regarding the mean intensity of parasitisation, it was much higher in Galicia (90.45 ± 134.96), where we found the most parasitised and documented animal to date in the province of Coruña, with 927 larvae. The communities of Asturias, País Vasco and Cantabria had a larval intensity of 48.6 ± 40.27, 38.11 ± 57.65 and 30.24 ± 34.19, respectively. These differences were statistically significant between Galicia and Cantabria and Galicia and País Vasco (*p* < 0.01) (Table 2).

In Galicia County, the presence of *Cephenemyia* stands out in all the sampled municipalities of Coruña, Orense and Pontevedra. In the province of Lugo, which was the most sampled, it was present in 92.86% (39/42) of the sampled municipalities except Begonte, Guitiriz and Valadouro.

In Asturias County, the presence of *Cephenemyia* stands out in all the sampled municipalities (4/4).

Cantabria County was the province in northern Spain with the lowest larval prevalence and intensity.

In País Vasco County, the presence of *Cephenemyia* stands out in all the sampled municipalities of Álava and Guipúzcoa, being absent only in one municipality.

In the Portuguese districts, we found a higher prevalence in the District of Vila Real, followed by Bragança. In Viana de Castelo, no positive animal was found, although the sample contained only two specimens. Regarding larval intensity, it was also higher in Vila Real.

Regarding the sex of the host, the prevalence was higher in females (77.18 ± 6.76% (95% IC)) than in males (67.16 ± 6.46 (95% IC)) and was statistically significant. If we remove only Portuguese animals, where almost all the animals were males (47/49), and we return to calculate the prevalence, this continued to be higher in females (78.23 ± 6.69 (95% IC)) than males (75.16 ± 6.78 (95% IC), but the differences were no longer statistically significant (*p* > 0.05). We also analysed whether there would be differences within the Spanish autonomous communities themselves. Only the province of Asturias was discarded due to its low number of samples and predominance of males (6/7). In all communities (Cantabria, Galicia and País Vasco), the prevalence was higher in females than in males, although it was only statistically significant in Cantabria. Regarding the larval intensity in this case, it was observed that it was greater in males (68.81 ± 120.83) than in females (56.85 ± 87.97); however, the differences were not statistically significant. When we calculated the intensities by autonomous communities, they were only higher in males in Cantabria and País Vasco and in females in Galicia, although it was only statistically significant in País Vasco (Table 3).

Regarding the age of the host, we saw a higher prevalence in old animals (78.26 ± 12.05% (95% IC)), followed by young animals (73.39 ± 7.81% (95% IC)) and adults (68.30 ± 6.76% (95% IC)). However, the differences were not statistically significant. On the other hand, the average number of larvae observed in roe deer from northern Spain and Portugal was much higher in young animals (109.76 ± 148.59), followed by old (33.64 ± 34.21) and adult (32.22 ± 39.01) animals. The results are statistically significant (*p* < 0.01) between young animals and adults and old roe deer without differences between adults and old animals (*p* > 0.05).

## 4. Discussion

It is important to monitor and try to establish effective control measures since, in certain areas in the northern peninsula where the prevalence of this disease is high, roe deer densities have experienced a decrease linked to a combination of several factors [26,27,28]. The presence of a high parasite load weakens animals, with some having many problems breathing and feeding. This makes these animals more immunosuppressed, increasing the risk of suffering from other diseases. Also, the animals with very high loads of mature larvae can die from drowning or starvation or otherwise be more susceptible to being prey to a predator (e.g., a dog, fox, wolf, etc.). The presence of deer in the areas where roe deer live causes a displacement of these from their territories. Finally, the considerable increase in wolf packs in all the autonomous communities studied in the last decade may influence the decline since a large part of the prey in their diet are roe deer [22,29,30,31].

Our results show that the prevalence in northern Spain is much higher (76.64%) than that detected by other authors in that area. The prevalence obtained in this region to date is between 16.5 and 62% in necropsies [6,32,33,34] or 25 and 57% in the ELISAs of serums [8,35]. It was also lower in other areas, such as central Spain (14.1%) [36]. This shows that its prevalence has doubled in the last decade and that more than three out of every four roe deer are currently infected by *C. stimulator* in northern Spain.

Furthermore, the prevalence obtained in this century by different European studies carried out in the Czech Republic, Poland, Hungary and the Slovak Republic is more similar to that of the rest of the Spanish studies, with values between 13 and 45.04% [11,14,37,38,39,40]. Only one study carried out last century by Dudziński in Poland obtained a prevalence of 85%, which was higher than that of our study [41].

Among the autonomous communities, the prevalence was higher in Galicia (91.41%) and País Vasco (86.27%) compared to Cantabria (55.93%). This may be because the first cases in the north of the peninsula were detected in the northwest area of Asturias and the province of Lugo in Galicia, being much more established there and could have later spread to the east of the peninsula. Likewise, País Vasco borders France, a country in which a seroprevalence of 40% was already described in animals analysed between 1994 and 1995 [42], so it is not ruled out that since there is no physical barrier between countries, the disease could also have entered naturally from that country.

Regarding the range of larvae/heads (1–927) found in our study, it was much higher than that described (1–95) by other authors in Spain and other European countries [6,8,11,14,37,39]. Also, the average intensity of larvae per animal detected was 60.42 ± 101.06 (much higher than others described) compared to 19.67 ± 21 to 24.3 in Galicia and Asturias [9,35], 16.9 ± 22.47 in Galicia and Cantabria [6] and 35.2 ± 49.71 in central Spain [36]. These high larval intensities can pose a risk to the life of the animal since the presence of between 30 and 80 mature larvae limits the chances of survival of roe deer [13]. Furthermore, this may have had an impact on roe deer densities since although populations have experienced growth in number and distribution throughout the entire Iberian Peninsula [43], population declines have been described in specific areas of the northern provinces of the peninsula, where the density was very high [26,27,28,29]. This is also reflected in the decrease in the number of annual captures from the 2011/2012 season to the 2021/2022 season, decreasing from 827 to 409 in Cantabria, from 6655 to 3023 in Galicia and from 1040 to 41 in Asturias [29]. In addition, recently, an increase in the number of sighted cases of another myiasis in the northwest of Spain has also been described, such as antler myiasis by *Lucilia caesar* in the case of male roe deer. This myiasis can also cause high mortality and has sometimes been seen together with *Cephenemyia* affecting the same animal, thus reducing its chances of survival [44,45].

Concerning the autonomous communities, we see much higher parasitisation intensities in Galicia compared to Cantabria or País Vasco (*p* < 0.01), which may be because the parasite has been living in that region for a longer time, making reinfestations by different flies more possible. Likewise, this is the first epidemiological study on *C. stimulator* that has been carried out in Portugal since it was first described in 2021 [17]. This has allowed us to see that this case was not an isolated event, and although the prevalence is not as high (38.78%) as those found in the north of Spain (76.64%), it is higher than those found in Spain a decade ago (25–31.6%) [9,32,35].

Regarding larval intensity, it was 37.74 ± 36.84, a fairly high parasite load if we compare it with those found in other European countries of 8.9 larvae/roe deer in Hungary [14], 1.6–14 and 32 larvae/roe deer in Poland [38,41], 15.24 in Slovakia [40] and 16.9 ± 22.47, 19.67 ± 21 and 24.3 in Spain [6,9,35]. For this reason, the disease must be taken into account, and measures must be applied to reduce its impact on the Portuguese roe deer population; otherwise, it could find itself in the same situation in the future as the entire Cantabrian population. The District of Vila Real and Braganza borders the south of the province of Ourense in Galicia, which is the autonomous community with the highest prevalence and parasite load in Spain. The lack of a physical barrier between countries can mean that populations of infected Spanish roe deer can enter Portugal, favouring their dispersion throughout the country.

Taking sex into account, we observed that the prevalence in females (77.18%) was statistically higher than in males (67.16%). We decided to try removing the Portuguese animals in case the results could be influenced since they were almost all males (47/49) and the prevalence there had been much lower than that of northern Spain, but the prevalence was still higher in females, although, in this case, the differences were not statistically significant. When treating each autonomous community separately, a higher prevalence was also found in females than in males, being statistically significant in Cantabria. These results could have been influenced by the region in which the samples were taken since some places where negative males were obtained did not have data on females. Given our results, we think that sex is not an influential factor like age can be. These results coincide with other authors, such as Király and Egri [14], who also obtained a higher prevalence in females than males, although it was not statistically significant. However, they disagree with those observed by other authors, such as Dudziński [41], Kornaś et al. [11], Pajares [9] and Pajares et al. [33], which detected a higher prevalence in males.

Larval intensity was higher in males (68.81 ± 120.83) than in females (56.85 ± 87.97), which also coincides with Király and Egri [14] and Martínez-Calabuig [6], although it differs from Pajares [9], who detected greater intensity in females. It was only statistically higher in males from País Vasco, although we believe this could have been influenced by several male specimens under one year of age, which had larval loads greater than a hundred.

Taking age into account, older animals presented the highest prevalence (78.26%), followed by young animals (73.39%) and adults (68.30%). These results coincide with those of many other authors [6,11,39], who detected higher prevalences in older animals. This may be because they are more likely to be exposed to the parasite over time, while roe deer kids in the first months of life can reduce their exposure to larviposition because they spend much of their time hidden among the vegetation. However, other authors detected higher prevalence in young animals [14,33], which was attributed to a lower immune response or lower defensive capacity against the flight of flies.

Regarding the parasite load, this was statistically higher in younger animals (109.76 ± 148.59) than in old animals (33.64 ± 34.21) and adults (32.22 ± 39.01). These results coincide with various authors who obtained higher values in young animals [6,11,36] than in older animals. This could be explained by the greater probability that younger roe deer leave the territory of their birth, making them more susceptible to being attacked by having to wander and go out to more open areas in search of a new home. As is the case with other oestrids, such as *Hypoderma* spp. (Latreille), a certain protective immune response may be generated so that first-infected animals harbour a greater number of larvae than reinfested ones, which may see the number of larvae reduced [46]. Likewise, those roe deer that have already been parasitised at some point can develop strategies to avoid being infected again, such as going out to open spaces less, especially during the hours of greatest fly flight activity.

## 5. Conclusions

Our results confirm that *C. stimulator* is widely spread in the north of the Iberian Peninsula in almost three-quarters of the analysed roe deer (71.39%). Furthermore, this decline in roe deer populations in specific areas of the northern peninsula may be largely caused by the high prevalence and larval intensity of this parasite. On the other hand, this is the first epidemiological study carried out on this parasite in the north of Portugal, and the prevalence obtained indicates that *Cephenemyia* is expanding and that in the coming years, it may experience the same growth that has already been suffered in the neighbouring country. Likewise, it has been seen that the youngest animals are those that have a greater parasite load, which can favour the spread of the disease since they are the most likely to be displaced from the territory in which they were born. Therefore, a specific management and training program for hunters is necessary to detect, monitor and implement measures to control and mitigate parasitosis.

## Figures and Tables

**Table 1:** Sampled roe deer organised by region, age and sex.

Factor	Category	No.
Region	Galicia	128
	Principado de Asturias	7
	Cantabria	118
	País Vasco	51
	Braganza	37
	Vila Real	10
	Viana do Castelo	2
Age	Young (<2 yr)	124
	Adult (2–6 yr)	183
	Old (>6 yr)	46
Sex	Female	149
	Male	204

**Table 2:** Prevalence and larval intensity in the different study areas.

Country	Study Area	Prevalence (95% CI)	Larval Intensity (SD)
Spain	Galicia	91.41 (4.87)	90.45 (134.96)
	País Vasco	86.27 (9.54)	38.11 (57.65)
	Principado de Asturias	85.71 (28)	48.6 (40.27)
	Cantabria	55.93 (9)	30.24 (34.19)
	Total	76.64 (4.76)	62.27 (104.40)
Portugal	Vila Real	60 (32.01)	52.17 (39.51)
	Bragança	35.14 (15.59)	31.08 (35.12)
	Viana do Castelo	-	-
	Total	38.78 (13.78)	37.74 (36.84)
Total		71.39 (4.72)	60.42 (101.06)

**Table 3:** Relationship of the influence of sex with the prevalence and larval intensity. F = female; M = male. * means the results were statistically significant.

Study Area	Prevalence	Larval Intensity
Total	F > M (*p* < 0.05) *	M > F (*p* > 0.05)
Spain	F > M (*p* > 0.05)	-
Cantabria	F > M (*p* < 0.05) *	M > F (*p* > 0.05)
Galicia	F > M (*p* > 0.05)	F > M (*p* > 0.05)
País Vasco	F > M (*p* > 0.05)	M > F (*p* < 0.05) *

## Data Availability

According to the General Regulation on Data Protection (GRDP) regulated by Law 59/2019, all data treated within the scope of this paper are confidential.
